# Pilot study of circulating cell-free mitochondrial DNA in relation to brain structure in youth bipolar disorder

**DOI:** 10.1186/s40345-024-00334-x

**Published:** 2024-06-14

**Authors:** Suyi Shao, Yi Zou, Kody G. Kennedy, Mikaela K. Dimick, Ana C. Andreazza, L. Trevor Young, Vanessa F. Goncalves, Bradley J. MacIntosh, Benjamin I. Goldstein

**Affiliations:** 1 Centre for Youth Bipolar Disorder, Centre for Addiction and Mental Health, Toronto, ON Canada; 2 Department of Pharmacology & Toxicology, Temerty Faculty of Medicine, University of Toronto, Toronto, ON Canada; 3 Department of Psychiatry, Temerty Faculty of Medicine, University of Toronto, Toronto, ON Canada; 4 Campbell Family Mental Health Research Institute, Centre for Addiction and Mental Health, Toronto, ON Canada; 5 Heart and Stroke Foundation Canadian Partnership for Stroke Recovery, Sunnybrook Research Institute, Toronto, ON Canada; 6 Department of Medical Biophysics, Temerty Faculty of Medicine, University of Toronto, Toronto, ON Canada; 7 Hurvitz Brain Sciences Program, Sunnybrook Health Sciences Centre, Toronto, ON Canada

**Keywords:** mtDNA, Bipolar disorder, Mitochondria, Magnetic resonance imaging, Youth

## Abstract

**Background:**

Mitochondrial dysfunction is implicated in the neuropathology of bipolar disorder (BD). Higher circulating cell-free mitochondrial DNA (ccf-mtDNA), generally reflecting poorer mitochondrial health, has been associated with greater symptoms severity in BD. The current study examines the association of serum ccf-mtDNA and brain structure in relation to youth BD. We hypothesized that higher ccf-mtDNA will be associated with measures of lower brain structure, particularly in the BD group.

**Methods:**

Participants included 40 youth (BD, *n* = 19; Control group [CG], *n* = 21; aged 13–20 years). Serum ccf-mtDNA levels were assayed. T1-weighted brain images were acquired using 3T-MRI. Region of interest (ROI) analyses examined prefrontal cortex (PFC) and whole brain gray matter, alongside exploratory vertex-wise analyses. Analyses examined ccf-mtDNA main-effects and ccf-mtDNA-by-diagnosis interaction effects controlling for age, sex, and intracranial volume.

**Results:**

There was no significant difference in ccf-mtDNA levels between BD and CG. In ROI analyses, higher ccf-mtDNA was associated with higher PFC surface area (SA) (β = 0.32 *p* < 0.001) and PFC volume (β = 0.32 *p* = 0.002) in the overall sample. In stratified analyses, higher ccf-mtDNA was associated with higher PFC SA within both subgroups (BD: β = 0.39 *p* = 0.02; CG: β = 0.24 *p* = 0.045). Higher ccf-mtDNA was associated with higher PFC volume within the BD group (β = 0.39 *p* = 0.046). In vertex-wise analyses, higher ccf-mtDNA was associated with higher SA and volume in frontal clusters within the overall sample and within the BD group. There were significant ccf-mtDNA-by-diagnosis interactions in three frontal and parietal clusters, whereby higher ccf-mtDNA was associated with higher neurostructural metrics in the BD group but lower neurostructural metrics in CG.

**Conclusions:**

Contrasting our hypothesis, higher ccf-mtDNA was consistently associated with higher, rather than lower, regional neuralstructural metrics among youth with BD. While this finding may reflect a compensatory mechanism, future repeated-measures prospective studies evaluating the inter-relationship among ccf-mtDNA, mood, and brain structure across developmental epochs and illness stages are warranted.

## Introduction

Bipolar disorder (BD) is a severe psychiatric disorder affecting approximately 1–3% of youth, an age group in which the manifestations of BD are particularly severe (Goldstein et al. 2017). The disorder is characterized by recurrent episodes of mania and depression, which are generally accompanied by pathological increases and decreases in energy, respectively (Jeong et al. 2020). Relatedly, mitochondrial dysfunction has been implicated in the etiology of BD (Chang et al. 2003; Kato et al. 2001; Munakata et al. 2004; Shao et al. 2008). Mitochondria are the energy producing organelles in cells and key players in the cerebral metabolism and energetics (Cuperfain et al. 2018; Giménez-Palomo et al. 2021). Magnetic resonance spectroscopy (MRS) findings in adults and youth with BD (Chang et al. 2003; Deicken et al. 2003; Kato et al. 1995; Stork and Renshaw 2005), and mitochondrial morphology abnormalities in fibroblasts from BD adults (Marques et al. 2021), suggest that abnormal mitochondrial morphology is linked to altered energy metabolism in the progression of BD (Marques et al. 2021).

Mitochondria have their own genome (mtDNA) which replicates independently from nuclear DNA. The mitochondrial genome encodes 2 ribosomal RNA, 22 transfer RNA (tRNA), and 13 electron transport chain (ETC) subunits (Kato 2001), all of which are related to oxidative phosphorylation (Kazuno et al. 2009). Under stress, fragments of mtDNA are released by the cells (i.e. circulating cell-free mtDNA – ccf-mtDNA) into the systemic circulation through both active (e.g. in microvesicles or in mitochondria-derived vesicles) and passive processes (e.g., apoptosis, necrosis) (Rostami et al. 2020; Jahr et al. 2001; Choi et al. 2005; Suzuki et al. 2008; De Gaetano et al. 2021). ccf-mtDNA concentrations in adult studies showed a wide range of values (Kageyama et al. 2018, 2022; Stertz et al. 2015). A group has shown that levels of ccf-mtDNA in adults with BD were lower in comparison to the controls (Kageyama et al. 2018), and that the ccf-mtDNA levels did not differ significantly between remitted and depressed BD patients (Kageyama et al. 2022). Another group has reported non-significant but higher ccf-mtDNA levels in BD patients in comparison to healthy controls (Stertz et al. 2015). Increased levels of ccf-mtDNA (from baseline to follow-up visits) or higher levels in cases in comparison with controls are a proxy of poorer mitochondrial health (Cordeiro et al. 2023), reflecting a fraction of the mtDNA that is released and extrusion of mitochondria during cell injury or stress (Jeong et al. 2020; Lindqvist et al. 2018). Nevertheless, increased levels of ccf-mtDNA is also reported to have beneficial characteristics, as it reflects intracellular communication, rescue of mitochondrial biogenesis pathways (Nicolás-Ávila et al. 2020), and clearance of damaged mitochondria (Davis et al. 2014).

Research focused on ccf-mtDNA in relation to psychopathology is emerging, as reflected by the following findings: elevated ccf-mtDNA levels were correlated with recent suicide attempt (Lindqvist et al. 2016), major depressive disorder (MDD) (Lindqvist et al. 2018), and acute psychosocial stress in healthy individuals (Trumpff et al. 2019). In adults with BD, higher ccf-mtDNA levels were associated with psychosis (Kageyama et al. 2022). A prior study from our group, based on an overlapping sample, found that higher ccf-mtDNA levels were significantly associated with more severe depression symptoms and higher lactate levels (another index of mitochondrial dysfunction) among youth with BD (Jeong et al. 2020). While the levels of ccf-mtDNA were 32.5% higher in youth with BD vs. CG, it was a non-significant difference. Nonetheless, the results of these studies indicate the value of further examining ccf-mtDNA in relation to BD (Jeong et al. 2020).

Abnormal brain structure has been reported in some mitochondrial disorders, as demonstrated by magnetic resonance imaging (MRI) studies (Barragán-Campos et al. 2005; Bianchi et al. 2007; Lerman-Sagie et al. 2005). Lower cortical and subcortical gray matter (GM) volume (Evangelisti et al. 2022) and lower cortical thickness (Haast et al. 2018) were observed in mitochondrial myopathy, encephalopathy, lactic acidosis, and stroke-like episodes (MELAS), a mitochondrial encephalopathy caused by A3243G mtDNA mutation (Bianchi et al. 2007). Another study also found lower GM volume in both MELAS patients and A3243G carriers (Tsujikawa et al. 2016). Meta-analysis of structural neuroimaging studies have identified lower regional GM volume and cortical thickness for BD relative to controls in a number of regions including the bilateral insula, superior temporal gyrus, and anterior cingulate cortex (Hibar et al. 2018; Wise et al. 2017). In addition, the ENIGMA subgroup analysis with 411 youth/young adults with BD found lower right supramarginal gyrus cortical thickness and left insula surface area (SA) in BD compared to the CG (Hibar et al. 2018). There is, however, a gap in knowledge regarding mitochondrial dysfunction, and levels of ccf-mtDNA in relation to brain structure in youth with BD.

The current study aims to examine the association between ccf-mtDNA levels and brain structure in relation to youth BD. We focused region-of-interest (ROI) analyses on the prefrontal cortex (PFC) and whole brain GM. In addition to BMI, we opted to include depressive and manic symptom severity (current, and most severe in lifetime), and duration of illness individually as covariates to examine the effects of illness burden on the association. Given the dearth of studies we also undertook a data-driven approach, examining GM SA, cortical thickness, and volume in vertex-wise analyses. We conducted stratified analyses with the BD and CG groups, followed by formal testing of interactions. We hypothesized that higher ccf-mtDNA levels would be associated with lower cortical GM SA, cortical thickness and volume in the overall sample. We also examined the evidence for an interaction effect, whereby there would be stronger associations of higher ccf-mtDNA levels with lower brain structure metrics in BD vs. CG.

## Methods

### Participants

This current study included 40 English-speaking youth between the ages of 13–20 years. Of these participants, 19 had a diagnosis of BD (type I, II, or not otherwise specified [NOS]) and 21 were in the CG. BD participants were recruited from a tertiary subspecialty youth BD clinic at an academic health science centre in Toronto, Ontario. All participants provided informed consent and had no pre-existing cardiac, inflammatory, and/or autoimmune illness, infectious illness in the past 14 days, substance dependence in the past three months, or contraindications to MRI. In addition, all participants were not taking hyperglycemic, anti-hypertensive, anti-platelet, anti-lipidemic, or daily anti-inflammatory medications. CG participants were primarily recruited via community advertisements. CG participants did not have any lifetime major psychiatric disorders (e.g., BD, MDD, psychosis) or alcohol/drug dependence, or first- or second-degree family history of BD or psychotic disorders. CG participants were also excluded if they had other psychiatric disorders and/or exposure to psychiatric medications in the past 3 months. Written informed consent was obtained from the participants and from their respective parent/guardian prior to the study procedures. All procedures were approved by the research ethics boards at Sunnybrook Health Sciences Centre and at Centre for Addiction and Mental Health (CAMH).

### Psychiatric and anthropometric measures

The Schedule for Affective Disorders and Schizophrenia for School Age Children, Present and Life Version (K-SADS-PL) (Kaufman et al. 1997), which is a semi-structured diagnostic interview, was employed to evaluate psychiatric diagnoses, treatment, and mood symptoms for all participants. The Diagnosis and Statistical Manual of Mental Disorders, 4th Edition criteria (DSM-IV) was used for the clinical diagnoses of BD-I and BD-II as the current sample was recruited between 2012 and 2018, whereas the DSM-5 version of the K-SADS-PL was not available until December 2016. The K-SADS Depression Rating Scale (DRS) and Mania Rating Scale (MRS) (Axelson et al. 2003; Chambers et al. 1985) were used to assess related mood symptom severity scores. Diagnosis of BD-NOS was based on operationalized criteria from the Course and Outcome of Bipolar Illness in Youth (COBY) study for duration of symptoms (minimum 4 h/day) and number of hypomanic days (minimum 4 in lifetime), while retaining DSM-5 symptom count requirements (i.e. 3 symptoms when elation was the primary symptom, 4 symptoms when irritability was the primary symptom) (Birmaher et al. 2009). All diagnoses and symptom ratings were confirmed by a licensed child and adolescent psychiatrist. Age of BD onset was the age at which the participant first experienced an episode of hypomania or mania that affected functioning, or met diagnostic criteria for BD-NOS. The Family History Screen was used to ascertain family psychiatric history for all first- and second- degree relatives (Weissman et al. 2000). Information regarding psychotropic medication use, and tobacco use were collected during the K-SADS-PL interview. Socioeconomic status was determined using the Hollingshead Four-factor Index (Hollingshead 1975). Tanner stage (1–5 stage scale) was determined using Pubertal Developmental Scale (Petersen et al. 1988). Weight was measured using a Tanita digital scale, and height was measured using a wall-mounted stadiometer. Body mass index (BMI) was calculated as weight in kilograms divided by the square of height in meters. Duration of illness in years was calculated by subtracting the age of onset of BD from the current age.

### Phlebotomy and biochemical assays

Blood samples were collected into a serum-separating tube via antecubital venipuncture between 8:00AM and 12:00PM. All participants were required to fast for 10 hours prior to the blood draw. Participants were required to abstain from using tobacco, alcohol, or any illicit drugs for 24 hours before their visit. Within 1 hour or less after the collection, blood samples were spun at 10,000 g and serum was aliquoted and stored at -80 °C until the day of biomarker analysis.

QIAamp DNA Mini Kit (Qiagen; Venlo, Netherlands) was used for circulating cell-free DNA (ccfDNA) extraction according to the manufacturer’s protocol using spin columns. 50 µL of serum was used for the collection of ccfDNA and 100 µL of UltraPure distilled water free of DNAse and RNAse (Invitrogen; CA, USA) was used to elute ccfDNA from the column. An estimated value of the absolute concentration of ccf-mtDNA, in copies/uL, was determined using quantitative polymerase chain reaction (qPCR) by comparing against a standard curve. Primer (Integrated DNA Technologies; IA, USA) was designed to amplify mitochondrial encoded NADH: Ubiquinone Oxidoreductase Core Subunit 1 (MT-ND1) gene, a gene that is unique to the mitochondrial genome. The forward primer sequence for MT-ND1 was CCCTAAAACCCGCCACATCT and the reverse primer sequence for MT-ND1 was GAGCGATGGTGAGAGCTAAGGT. A standard curve was prepared using a purchased oligonucleotide of the PCR product (Integrated DNA Technologies; IA, USA) at a known quantity, followed by serial dilution into concentrations ranging from 100 copies/uL to 1.0 × 10^8^ copies/uL. qPCR was carried out using a total reaction volume of 20 µL. qPCR reaction was composed of 10 µL of 2X SensiFast SYBR with No-ROX (Bioline; London, UK), 1 µL of 10 µM forward primer, 1 µL of 10 µM reverse primer, 2 µL of ultra-pure distilled water, and 6 µL of ccfDNA. Each reaction was replicated three times on Bio-Rad’s C1000 Thermal Cycle CFX96 Real-Time System (CA, USA). The qPCR procedures are as follows: initial denaturation and hot-start at 95 °C for 3 min, followed by 40 cycles that consist 95 °C for 10 s, 60 °C for 20 s, and a fluorescence measurement. In the end, a melting curve analysis was performed by measuring from 65 °C to 95 °C, increasing at 0.5 °C for every 5 s proceeding with a fluorescent read. Inter-assay variability was evaluated as randomly selected samples of eluted ccf-mtDNA were pooled randomly at equal volumes and were run on all plates. The intra-assay variability of the technical replicates was 3.0% and the inter-assay variability between qPCR plates was 9.16%. The experimenter was blinded to the diagnosis of the participants.

### Magnetic resonance imaging acquisition and processing

Structural images of the brain were collected on a 3 Tesla Philips Achieva MRI scanner (Philips Medical Systems, Best, Netherlands). Acquisition of MRI data used the body coil and 8-channel head coil for signal transmission and receiving. The high-resolution fast-field echo (FFE) T1-weighted images were acquired with the following parameters: repetition time (TR) 9.5 milliseconds (ms), echo time (TE) 2.3ms, inversion time (TI) 1400ms, spatial resolution 0.94 × 1.17 × 1.2 mm, acquisition matrix of 256 × 164 × 140, field of view (FOV) 240 × 191 mm, 8° flip angle, and scan duration 8 min and 56 s.

Three-dimensional (3D) reconstruction of the T_1_-weighted images was performed using FreeSurfer version 6.0 software. Quality control steps include head motion correction and other artifacts. Further processing steps of the 3D images include removal of non-brain tissue, automated skull stripping, field inhomogeneity correction, automated segmentation classified subcortical structures, tessellation of the cortical white and GM boundary, and topology correction (Dale et al. 1999; Fischl et al. 1999a, b, 2001, 2002, 2004b; Fischl & Dale 2000; Ségonne et al. 2004, 2007; Sled et al. 1998). Finally, the image was inflated and mapped to a spherical atlas, allowing anatomical alignment of the brain. Cortical parcellation was then completed based on the Desikan-Killiany (DK) probabilistic atlas to label 34 gyral regions of interest per hemisphere (Fischl et al. 2004a). Three independent raters visually inspected the processed images for quality (e.g., artifacts, and contrast between white matter and GM) and parcellation accuracy (e.g., correctly labeled structures) using a 0–3 scoring scale; poor-accuracy parcellations were edited. Images with poor parcellation or poor quality were excluded from the dataset. The average time between blood collection and MRI acquisition was 89.6 ± 78.0 days.

### Statistical analysis

Statistical analyses were performed using the SPSS statistic software version 26 (IBM; NY, USA) for clinical and demographic variables. The normality of all continuous variables were assessed using the Shapiro-Wilks test. The equal variance assumptions of all continuous variables were checked using Levene’s test. Between-group differences in demographic and clinical characteristics were assessed using independent-samples *t*-tests, Mann-Whitney *U*-tests, and Kruskal-Wallis test for continuous and ordinal variables or chi-square tests for categorical variables as appropriate. Effect sizes are reported as Cramer’s V (*V*) or Cohen’s d (*d*) or eta-squared (η^2^).

We defined cortical ROIs using annotations from the DK atlas. The PFC consisted of rostral middle frontal gyrus, caudal middle frontal gyrus, rostral anterior cingulate cortex, caudal anterior cingulate cortex, orbitofrontal cortex, superior frontal gyrus, inferior frontal gyrus, and frontal pole. Volume and SA for each ROI were calculated by adding up the values for each annotation within an ROI for both hemispheres. Cortical thickness was calculated proportionally to the SA. The associations of ccf-mtDNA with ROIs (i.e. PFC and whole brain GM) were tested using a General Linear Model (GLM) with age, sex, and intracranial volume (ICV) as covariates within the overall sample and within the BD and CG groups. ICV was not included as a covariate in the model for cortical thickness analyses (Barnes et al. 2010). Between-group differences (interaction effects) in the association of ccf-mtDNA levels with ROIs were also examined. Bonferroni correction was used to correct for multiple ROI comparisons (dividing the significance level by the number of ROIs, α = 0.05/2 = 0.025). 

For the whole-brain vertex-wise exploratory analyses, we smoothed the brain surfaces of participants using a 10 mm kernel of full-width at half-maximum. Volumetric, SA, and cortical thickness data from each participant were then mapped to the canonical template. Vertex-wise analyses were performed using the aforementioned GLMs from the ROI analyses. The significance level was set at *p* < 0.05 and correction for multiple comparisons was performed with permutation testing within the FreeSurfer package (Fischl 2012). Cluster-wise *p*-values (cwp) were then calculated as the probability of seeing a cluster of the given size during the permutations and were reported for each significant cluster. Post-hoc analyses were conducted for regions that revealed significant ccf-mtDNA main-effect and/or ccf-mtDNA-by-diagnosis interaction effect to investigate within-group associations of ccf-mtDNA.

Lastly, we undertook a series of sensitivity analyses by adding BMI, depressive and manic symptom severity (current, and most severe in lifetime), and duration of illness as a covariate individually in addition to the covariates used previously. In the post-hoc analyses and sensitivity analyses for the vertex-wise findings each significant cluster was treated as an ROI.

## Results

### Demographic and clinical characteristics

Demographic characteristics are summarized in Table 1. This study included 40 youth, 19 with BD (6 BD-I, 10 BD-II, 3 BD-NOS), and 21 CG participants. There were no significant differences in age, sex, or race between BD and CG. Compared to the CG (mean = 21.2 ± 2.6), the BD group (mean = 23.6 ± 2.9) had greater BMI (*p* = 0.01 *d* = 0.87). Clinical characteristics are summarized in Table 2.

**Table 1:** Demographic characteristics

	BD (*n* = 19)	CG (*n* = 21)	t^a^/χ^2b^/H^c^	*p*-Value	Effect size
Age	17.4 ± 1.7	16.3 ± 1.7	1.98^a^	0.06	0.65^d^
Sex (n, % Female)	10 (53)	10 (48)	0.10^b^	0.75	0.05^v^
Race (n, % Caucasian)	15 (79)	16 (76)	2.27^b^	0.81	0.24^v^
BMI (adjusted)	23.6 ± 2.9	21.2 ± 2.6	2.74^a^	0.01	0.87^d^
Tanner Stage			0.09^c^	0.77	0.02 ^η2^
(n, % stage 3)	2 (11)	1 (5)			
(n, % stage 4)	10 (53)	12 (57)			
(n, % stage 5)	7 (37)	8 (38)			
ccf-mtDNA	2929.5 ± 2124.2	2257.2 ± 1452.8	1.18^a^	0.25	0.37^d^

Note: Results are reported as mean ± standard deviation (SD) or percentage (%) unless otherwise specified. BD = Bipolar disorder; CG = Control group

^a^ = t-test, ^b^ = Chi-square Test, ^c^ =Kruskal-Wallis test

Effect size was reported in as Cohen’s d = *d* for t-test, Cramer’s V = *V* for Crosstab, eta-squared = η^2^ for Kruskal-Walllis test

**Table 2:** Clinical characteristics

	BD (*n* = 19)
BD-I	6 (32%)
BD-II	10 (53%)
BD-NOS	3 (16%)
Age of BD onset (years)	14.1 ± 2.7
**Lifetime Clinical Characteristics**	
Psychosis	7 (37%)
Suicide attempts	3 (16%)
Self-injurious behaviour	8 (42%)
Physical/ sexual abuse	2 (11%)
Psychiatric hospitalization	11 (58%)
Current depression score^a^	12.4 ± 10.1
Lifetime depression score^a^	28.0 ± 13.7
Current mania score^b^	8.8 ± 11.0
Lifetime mania score^b^	28.6 ± 12.2
Duration of illness (years)	3.3 ± 2.3
**Lifetime Comorbid Diagnoses**	
ADHD	10 (53%)
Anxiety disorder	13 (68%)
Number of anxiety disorders	1.1 ± 0.9
Conduct disorder	2 (11%)
Oppositional defiant disorder	5 (26%)
Substance use disorder	4 (21%)
Smoking	11 (58%)
**Current Medications**	
Second generation antipsychotics	12 (63%)
Lithium	4 (21%)
Non-SSRI antidepressants	0 (0%)
SSRI antidepressants	1 (5%)
Stimulants	3 (16%)
Any medication	15 (79%)
**Family Psychiatric History**	
Mania/hypomania	11 (58%)
Depression	14 (74%)
Anxiety	10 (53%)
ADHD	8 (42%)
Psychosis	3 (16%)
Substance use disorder	5 (26.3%)
Suicide attempt	6 (31.6%)

BD = Bipolar disorder; NOS = Not otherwise specified; ADHD = Attention deficit-hyperactivity disorder; SSRI = Selective serotonin reuptake inhibitor. Note: Results are reported in mean ± standard deviation (SD) or percentage (%) unless otherwise specified

Missing cases (n): Family Psychiatric History Depression (3); Family Psychiatric History Mania/hypomania (3); Family Psychiatric History Anxiety (3); Family Psychiatric History ADHD (3); Family Psychiatric History Psychosis (3); Family Psychiatric History Suicide Attempt (3)

^a^Depression scores based on Depression Rating Scale

^b^Mania scores based on Mania Rating Scale

### ccf-mtDNA region of interest analyses

There was no significant difference in ccf-mtDNA levels comparing subjects with BD and controls (BD = 2929.5 ± 2124.2, CG = 2257.2 ± 1452.8, *p* = 0.25). The association of ccf-mtDNA levels with ROI brain structure in the overall sample is presented in Table 3. Within the overall sample, higher ccf-mtDNA was associated with higher PFC SA (β = 0.32 *p* < 0.001) and volume (β = 0.32 *p* = 0.002). Within the BD group, higher ccf-mtDNA was associated with higher PFC SA (β = 0.39 *p* = 0.02) and higher PFC volume (β = 0.39 *p* = 0.046). Within the CG, higher ccf-mtDNA was also associated with higher PFC SA (β = 0.24 *p* = 0.045). After correction for multiple comparisons, higher ccf-mtDNA was still associated with higher PFC SA within the overall sample and BD, and higher PFC volume within the overall sample. After controlling for BMI, the PFC volume and SA findings within the whole sample and the PFC area finding within the BD group remained significant. No associations between ccf-mtDNA and cortical thickness were found in ROI analyses. There were no significant group-by-ccf-mtDNA interactions. All findings remained significant after controlling for current depressive and manic symptom severity but not after controlling for past depressive and manic symptom severity. After controlling for duration of illness, the PFC area findings within the BD group remained significant.

**Table 3:** Results of the association of ccf-mtDNA and the ccf-mtDNA-by-diagnosis interaction effects with brain structure in regions of interest

**Main-effects of ccf-mtDNA on Brain Structure**												
	**PFC** **area**		**PFC** **volume**		**PFC** **thickness**		**WB** **area**		**WB** **volume**		**WB** **thickness**	
	**β**	**p**	**β**	**p**	**β**	**p**	**β**	**p**	**β**	**p**	**β**	**p**
Overall Sample	0.32	< 0.001*	0.32	0.002*	0.09	0.58	0.13	0.06	0.14	0.07	0.15	0.31
BD	0.39	0.02*	0.39	**0.046**	0.04	0.87	0.25	0.05	0.25	0.10	0.10	0.71
CG	0.24	**0.045**	0.31	0.05	0.14	0.56	0.001	0.99	0.05	0.66	0.19	0.39
**ccf-mtDNA-by-Diagnosis Interaction Effects on Brain Structure**												
	**PFC** **area**		**PFC** **volume**		**PFC** **thickness**		**WB** **area**		**WB** **volume**		**WB** **thickness**	
	**β**	***p***	**β**	***p***	**β**	***p***	**β**	***p***	**β**	***p***	**β**	***p***
BD Vs. CG	0.04	0.69	0.002	0.98	-0.06	0.73	0.10	0.21	0.06	0.44	-0.05	0.77

PFC = Prefrontal cortex; WB = Whole brain; Significant group effects are bolded. *= Findings remain significant after correction for multiple comparisons

### ccf-mtDNA whole-brain vertex-wise analyses

Table 4 summarizes the clusters identified across all vertex-wise analyses.

**Table 4:** Results of the association of ccf-mtDNA and the ccf-mtDNA-by-diagnosis interaction effects with brain structure from vertex-wise analyses

**Main-effects of ccf-mtDNA on Brain Structure**															
**Diagnosis**	**Cortical Measure**	**Peak Cluster Region**	**Included Regions**	**Size of Cluster (mm** ^**2**^ **)**	**cwp**		**MNI X**			**MNI Y**		**MNI Z**		**β**	
Overall Sample	Area	Left superior frontal gyrus	Rostral anterior cingulate cortex, rostral middle frontal gyrus	4415	0.0001		-7.1			48.6		35.5		0.47	
		Right rostral middle frontal gyrus	Superior frontal and lateral orbitofrontal gyri	2795	0.0001		23.0			60.4		4.0		0.47	
	Volume	Left superior frontal gyrus	Rostral anterior cingulate cortex	2158	0.0001		-10.9			24.9		55.7		0.59	
BD	Area	Left superior frontal gyrus	Medial orbitofrontal and rostral middle frontal gyri, rostral anterior cingulate cortex, frontal pole	3432	0.0001		-7.3			48.2		34.8		0.67	
		Left precentral gyrus	Postcentral gyrus	1449	0.03		-48.1			2.3		23.2		0.63	
		Right superior frontal gyrus	Medial orbitofrontal gyrus, rostral anterior cingulate cortex	2403	0.0008		15.0			39.8		18.0		0.61	
		Right superior frontal gyrus	Rostral middle frontal gyrus, frontal pole	1417	0.0358		12.9			62.8		6.4		0.53	
	Volume	Left superior frontal gyrus	Rostral anterior cingulate cortex	1780	0.0001		-8.6			34.8		42.1		0.72	
		Left precentral gyrus	Postcentral gyrus	1698	0.0001		-39.9			0.7		27.5		0.68	
**ccf-mtDNA-by-Diagnosis Interaction Effects on Brain Structure**															
**Diagnosis**	**Cortical Measure**	**Peak Cluster Region**	**Included Regions**	**Size of Cluster (mm** ^**2**^ **)**		**cwp**		**MNI X**	**MNI Y**		**MNI Z**		**β-BD**		**β-CG**
BD vs. CG	Area	Left precentral gyrus	Postcentral gyrus	1751		0.0079		-56.5	-1.7		32.1		0.59		-0.38
	Volume	Left postcentral gyrus	Precentral gyrus	1418		0.0005		-51.3	-10.6		15.0		0.64		-0.45
		Left superior parietal lobule	Postcentral gyrus	874		0.0201		-25.1	-43.9		55.6		0.58		-0.61

cwp = cluster wise *p*-value; MNI = Montreal Neurological Institute and Hospital

#### Main-effects

**Fig. 1:**
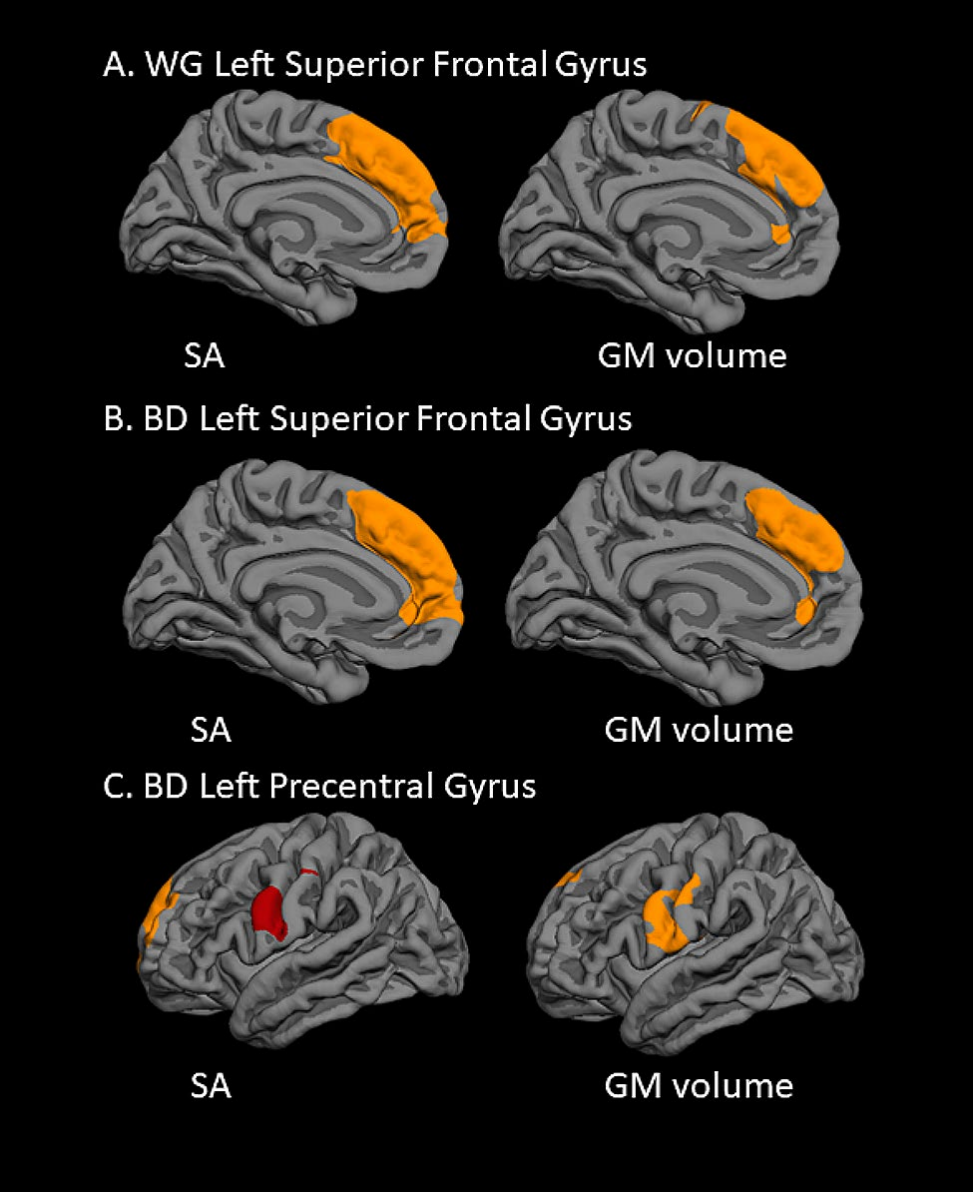
Overlapping regions in which ccf-mtDNA was associated with both the SA (left column) and the GM volume (right column). (**A**) Within whole group (WG), higher ccf-mtDNA was significantly associated with higher left superior frontal gyrus SA (β = 0.47 *p* < 0.001) and left superior frontal gyrus GM volume (β = 0.59 *p* < 0.001). (**B**) Within BD, higher ccf-mtDNA was significantly associated with higher left superior frontal gyrus SA (β = 0.67 *p* < 0.001) and left superior frontal gyrus GM volume (β = 0.72 *p* < 0.001). (**C**) Within BD, higher ccf-mtDNA was significantly associated with higher left precentral gyrus SA (β = 0.63 *p* = 0.003) and left precentral gyrus GM volume (β = 0.68 *p* = 0.001)

Within the overall sample, higher ccf-mtDNA was associated with higher whole-brain SA in two clusters, one within the left superior frontal gyrus and another within the right rostral middle frontal gyrus. Additionally, higher ccf-mtDNA was associated with higher volume in a left superior frontal gyrus cluster. Within the BD group, 6 clusters were identified: 4 for SA (located in left superior frontal gyrus, left precentral gyrus, and right superior frontal gyrus) and 2 for volume (located in left superior frontal gyrus and left precentral gyrus). Specifically, in the overall sample, SA and volume findings overlapped within the left superior frontal gyrus cluster. Similarly, in the BD group, SA and volume findings overlapped within the left superior frontal gyrus cluster and the left precentral gyrus cluster (Fig. 1). There were no significant clusters for the CG. Further controlling for BMI, depressive and manic symptom severity (current, and most severe in lifetime), and duration of illness did not change these findings.

#### Interaction effects

**Fig. 2:**
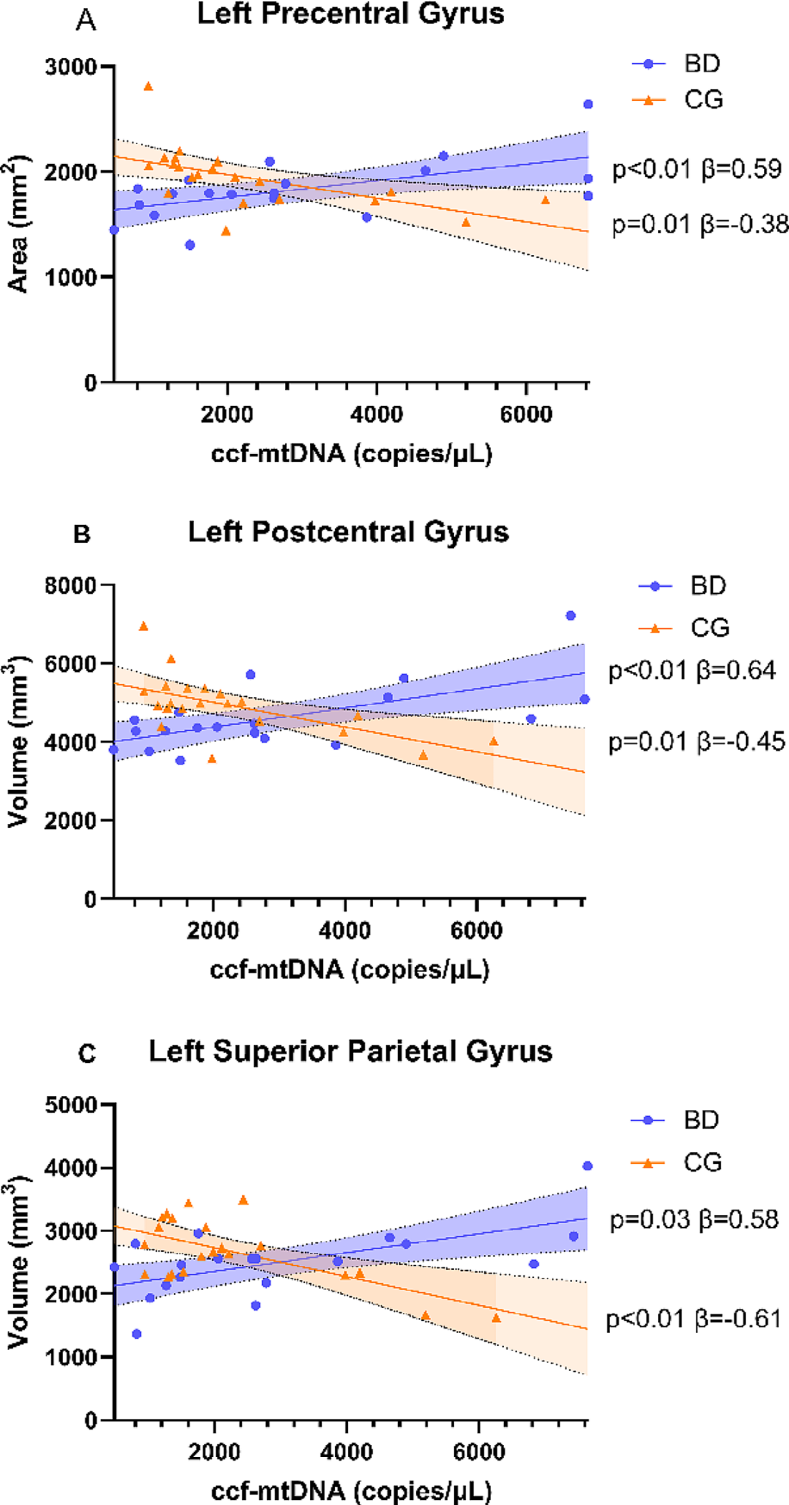
Regions with significant ccf-mtDNA-by-diagnosis differences identified from vertex-wise analyses. (**A**) ccf-mtDNA-by-diagnosis interaction effect on left precentral gyrus area. Within CG, higher ccf-mtDNA was significantly associated with lower SA (β=-0.38 *p* = 0.01). Within BD, higher ccf-mtDNA was significantly associated with higher SA (β = 0.59 *p* < 0.01). (**B**) ccf-mtDNA-by-diagnosis interaction effect on left postcentral gyrus volume. Within CG, higher ccf-mtDNA was significantly associated with lower volume (β=-0.45 *p* = 0.01). Within BD, higher ccf-mtDNA was significantly associated with higher volume (β = 0.64 *p* < 0.01). (**C**) ccf-mtDNA-by-diagnosis interaction effect on left superior parietal lobule volume. Within CG, higher ccf-mtDNA was significantly associated with lower volume (β=-0.61 *p* < 0.01). Within BD, higher ccf-mtDNA was significantly associated with higher volume (β = 0.58 *p* = 0.03)

Three significant clusters were identified for left precentral gyrus SA, left postcentral gyrus volume and left superior parietal lobule volume (Fig. 2). Post-hoc analyses revealed that for each cluster, higher ccf-mtDNA levels were significantly associated with higher neurostructural metrics in the BD group, whereas higher ccf-mtDNA levels were significantly associated with lower neurostructural metrics in the CG. Further controlling for BMI, depressive and manic symptom severity (current, and most severe in lifetime), and duration of illness did not change these findings.

## Discussion

The current study investigated the association between ccf-mtDNA and measures of brain structure in relation to youth BD. The major finding is that higher ccf-mtDNA levels were associated with higher regional GM volume and SA in both ROI analyses and whole brain vertex-wise analyses within the BD group and the overall sample. There were also significant ccf-mtDNA-by-diagnosis interaction effects in three clusters in frontal and parietal regions that mediate functions such as memory, attention, voluntary motor movements, and proprioception; in each case, higher ccf-mtDNA levels were associated with higher neurostructural metrics in the BD group and lower neurostructural metrics in the CG. The findings within the BD group are contradictory to our hypotheses, as they suggest potentially beneficial roles of ccf-mtDNA in BD.

ROI analysis revealed that higher ccf-mtDNA levels were associated with higher PFC GM volume within overall sample and within each subgroup, and with higher PFC SA within the overall sample and within the BD group. Post-mortem studies have found evidence of altered levels of mitochondrial gene methylation and protein levels (Andreazza et al. 2010, 2013; Clay et al. 2011; Shi et al. 2012; Wang 2007), reduced mitochondria size (Cataldo et al. 2010), and altered levels of antioxidant enzymes and proteins (Gawryluk et al. 2011a, b) in the PFC of patients with BD. Together, these findings suggest that mitochondrial dysfunction in PFC of individuals with BD and the subsequent oxidative damage may be related to neuron loss in the PFC of individuals with BD (Andreazza et al. 2013). Therefore, while higher levels of ccf-mtDNA reflects mitochondrial dysfunction, which is associated with neuronal loss in the PFC, the association of higher ccf-mtDNA with higher PFC volume and SA runs counter to our hypotheses. However, interestingly the ccf-mtDNA released through mitochondria-derived vesicles can act as intercellular messengers and rescue mitochondrial defects (Islam et al. 2012; Torralba et al. 2016). Thus, our result might suggest that ccf-mtDNA could act as an intercellular messenger, which potentially could activate mitochondrial biogenesis pathways.

Vertex-wise analyses revealed that higher ccf-mtDNA level was associated with higher SA and GM volume in the left superior frontal gyrus within the overall sample and within the BD group. The left superior frontal gyrus is a critical subregion of the PFC that processes higher cognitive functions, especially spatial working memory (du Boisgueheneuc et al. 2006), which is known to be impaired in BD (du Boisgueheneuc et al. 2006; Joseph et al. 2008; Li et al. 2013). In addition, higher ccf-mtDNA was associated with higher right superior frontal gyrus SA within the overall sample and with higher right superior frontal gyrus volume within the BD group. Right superior frontal gyrus is associated with control of impulsive response (Hu et al. 2016) and demonstrated reduced activation in pediatric BD during trials requiring response inhibition compared to the CG (Passarotti et al. 2010). Thus, our vertex-wise analyses might be considered as an expansion of our ROI analyses as they highlight specific anatomical segregation of PFC that corresponds with specialized certain brain functions (Goldman-Rakic 1996; Levy and Rakic 2000). Within the BD group, we identified another cluster in the left precentral gyrus where higher ccf-mtDNA levels were associated with higher SA and GM volume. Prior studies have found reduced precentral gyrus GM volume in adults with BD (Altamura et al. 2018; Gao et al. 2021). In addition to other functions, the precentral gyrus is involved in regulating impulsivity and inhibitory control, which are relevant in BD (Dimick et al. 2022).

Vertex-wise interaction analyses identified three clusters that span left precentral gyrus SA, left postcentral gyrus volume, and left superior parietal lobule volume. Notably, the left precentral gyrus SA cluster and the left postcentral gyrus volume cluster were overlapping. In this brain region, ccf-mtDNA was associated with higher GM SA and volume in the BD group and was associated with lower GM SA and volume in the CG. The precentral gyrus is responsible for executing voluntary movements, which is also impaired among individuals with BD (Hirjak et al. 2018; Kent et al. 2020; Lohr and Caligiuri 2006). The postcentral gyrus is on the lateral surface of the parietal lobe and contains the primary somatosensory cortex, a brain region responsible for emotion processing and regulation (Kropf et al. 2019). The superior parietal lobule is involved in executive function and spatial working memory (Cho and Goghari 2020; Koenigs et al. 2009; Wager and Smith 2003).

Neither the ROI analysis nor the vertex-wise whole brain analysis generated significant cortical thickness findings. SA and cortical thickness are distinct features of cortical structures with low genetic and environmental correlation (Panizzon et al. 2009; Winkler et al. 2010), and GM volume is the product of SA and cortical thickness. Cortical SA is determined by the number of the cortical columns (Pontious et al. 2008) and by neurodevelopmental alterations in regional gyrification (Hogstrom et al. 2013). On the other hand, cortical thickness represents the number of neurons within a cortical column and is affected by neuronal survival (Pontious et al. 2008). We speculate that ccf-mtDNA may be more closely related to regional gyrification as compared to neurotoxicity. Additionally, there is evidence from postmortem studies (Pakkenberg and Gundersen 1997) and studies of healthy participants (Im et al. 2008) that while cortical thickness increases slightly with increasing GM volume, SA increases greatly. Thus, the GM volume findings may be driven by SA. This is also supported by our vertex-wise whole-brain analysis, where the GM volume clusters largely overlap with the SA clusters (Fig. 1).

Our results indicate differential associatton of ccf-mtDNA with brain structure in youth with BD vs. CG, suggesting differential mechanisms underlying these associations. The discrepancy may reflect differential susceptibility to mitochondrial dysfunction-related oxidative stress (Zou et al. 2022) or differential effects/sources of ccf-mtDNA in youth with BD vs. CG. Apoptosis is the dominant mechanism by which ccf-mtDNA is released in healthy individuals (De Gaetano et al. 2021; Jahr et al. 2001; Rostami et al. 2020), which aligns with the finding that ccf-mtDNA levels were negatively associated with brain structure in CG. In contrast, the positive association of ccf-mtDNA with brain structure in BD may reflect the beneficial characteristics of ccf-mtDNA, namely intracellular communication, rescue of mitochondrial biogenesis pathways (Nicolás-Ávila et al. 2020), and clearance of damaged mitochondria (Davis et al. 2014). Another potential explanation for this unexpected finding is that ccf-mtDNA reflects overall mitochondrial dysfunction in the BD group (Cordeiro et al. 2023), which in turn elicits compensatory adaptations in the brain. We previously proposed that compensatory mechanisms may underlie the abnormally elevated regional cerebral blood flow (CBF) in youth with BD (Karthikeyan et al. 2019). Nonetheless, the current findings are observational and cross-sectional and were not designed to elucidate the mechanisms of the observed associations. Further prospective repeated-measures observational studies as well as mechanistic studies are warranted to gain further insights regarding mitochondrial dysfunction and compensatory mechanisms in relation to BD. Our results should be considered in light of several limitations. First, the cross-sectional study design does not provide inferences regarding the dynamic influence of ccf-mtDNA on brain structure across development. Second, we did not observe a significant between-group difference in ccf-mtDNA levels (BD = 2929.5 ± 2124.2, CG = 2257.2 ± 1452.8, *p* = 0.25), limiting the interpretability of the interaction effect. Third, the small sample precludes secondary analyses focused on important sources of variability, such as BD subtype and sex differences. Fourth, as we did not measure circulating cell-free nuclear DNA, this prevents the assessment of mitochondrial-to-nuclear circulating cell-free DNA, in turn limiting interpretations of present findings (Trumpff et al. 2021). Fifth, this study used one set of primers for the mtDNA quantification, as it was initiated prior to the contemporary approach of using two sets of primers, which optimizes the accurate detection of mtDNA (Trumpff et al. 2019). Finally, the blood samples were not always collected on the day of neuroimaging. The average duration between imaging and mtDNA collection was approximately three months, and it remains possible that time-related factors impacted current findings.

In summary, we showed that ccf-mtDNA is associated with brain structure, particularly SA, in frontal-parietal brain regions with known relevance to emotional regulation and neurocognitive domains that are central to BD. We also found preliminary evidence that ccf-mtDNA is differentially associated with brain structure in youth with BD vs. CG. Despite its limitations and observational design, this study highlights potential brain mechanisms through which mitochondrial dysfunction may impact BD. A key aspect of this study is its focus on youth with BD who are early in their course of illness and in the midst of a key neurodevelopmental epoch. As the current cross-sectional design does not reflect the dynamic relationship between ccf-mtDNA and brain structure, future longitudinal studies with larger samples are warranted. In addition, it will be important to incorporate participants from across the lifespan with different duration of illness and at different stages of illness to examine the relevance of ccf-mtDNA to progressive changes in brain structure during the course of BD. Finally, future studies are needed to identify subgroups in whom ccf-mtDNA may be particularly relevant.

## Data Availability

The datasets used and/or analysed during the current study are available from the corresponding author upon reasonable request. The data are not publicly available due to privacy or ethical restrictions.

## Abbreviations

ccf-mtDNA: Circulating cell-free mitochondrial DNA
BD: Bipolar disorder
CG: Control Group
ROI: Regions of interest
PFC: Prefrontal cortex
GM: Gray matter
SA: Surface area
